# Supplementary material for: Experimental study of high-flow and low-expansion backfill material
Source: PLoS One. 2020 Aug 14;15(8):e0236718. doi: 10.1371/journal.pone.0236718 (PMC7428165; doi:10.1371/journal.pone.0236718)
Supplement: Attachment — Submitted filename: Response to Reviewers.docx [file pone.0236718.s002.docx]

Dear Prof./Dr. Kedsarin Pimraksa:

Thank you very much for considering our manuscript. All authors would like to take this opportunity to express our gratitude to you and the anonymous reviewers for the valuable suggestions on our paper (Paper no: PONE-D-20-00990R1, Paper title: Experimental study of high-flow and low-expansion backfill material).

According to the comments, we have carefully revised the manuscript and made it easy to be understood. The revised manuscript is attached to this email, and questions are all answered one by one in the enclosed files. We hope the revised manuscript can meet these requests and to be accepted.

I look forward to hearing from your decision as soon as possible.

Best regards.

Cheng Wang, Chun Wang, Zuqiang Xiong, Yuli Wang, Yafeng Han

**To** **Academic Editor’s Evaluations:**

**Comment 1:** Inconsistent word: Author used both mineral powder and mine powder in many places.

**Response:** Thank you very much. As you suggested that we have changed the inconsistent word. **And the revised portions are marked in this manuscript.**

**Comment 2:** Missing of characterization explanation: Sample preparation for SEM analysis should be explained in experimental and methods.

**Response:** Thank you very much. According to your suggested that we have added the characterization explanation, **and the revised portions are marked in this manuscript.**

**The SEM sample was taken from the specimens in uniaxial compression test, the sample was made by 1cm*1cm. Then it was fixed on the metal plate with double-sided conductive adhesive, the metal plate was placed in a gold-spraying apparatus for gold spraying, drying and vacuuming, and finally placed in a SEM instrument for the experiment.**
